# Traumatization, Loneliness, and Suicidal Ideation among Former Prisoners of War: A Longitudinally Assessed Sequential Mediation Model

**DOI:** 10.3389/fpsyt.2017.00281

**Published:** 2017-12-12

**Authors:** Jacob Y. Stein, Liat Itzhaky, Yossi Levi-Belz, Zahava Solomon

**Affiliations:** ^1^I-CORE Research Center for Mass Trauma, Bob Shapell School of Social Work, Tel Aviv University, Tel Aviv, Israel; ^2^Department of Behavioral Sciences, Ruppin Academic Center, Emek-Hefer, Israel

**Keywords:** war captivity, posttraumatic stress disorder, loneliness, suicide, veterans, suicidal ideation, POWs, trauma

## Abstract

Although highly researched among veterans, the underlying mechanisms of suicidal ideation (SI) among former prisoners of war (ex-POWs), especially in the long-term, have rarely been investigated. Furthermore, while posttraumatic stress symptoms (PTSS) and loneliness have been individually associated with veteran SI, and both may be differentially implicated by captivity versus war traumas, the interplay between them has yet to be examined. Filling this gap, the current longitudinal study examined a hypothetical sequential model wherein war captivity, compared with combat-induced trauma, is implicated in worse PTSS, which is then implicated in worse loneliness and PTSS, which together may explain subsequent SI. Two groups of Israeli veterans of the 1973 Yom Kippur War, 163 ex-POWs and 185 matched non-captive veterans were assessed 18 (T1) and 30 (T2) years after the war. Analyses indicated that compared with war, captivity was implicated in worse PTSS, which was implicated in worse loneliness, and these worked in tandem to implicate SI. Loneliness, however, was not directly affected by the type of trauma, nor was its relation to SI linked to its implication in subsequent PTSS. These results may inform future research and clinical practice as the study underscores the importance of both PTSS and loneliness in ex-POWs’ long-term SI.

## Introduction

The study of suicide among military personnel and veterans is gaining prominence, as suicide rates among this population seem to be reaching epidemic levels worldwide [e.g., Ref. (1, 2)]. Suicidal ideation (SI), the contemplation of taking one’s own life, is a major risk factor for suicidal behaviors, suicide attempts, and suicide completions (3). Epidemiological reviews indicate that among the general population, 90% of unplanned and 60% of planned first suicide attempts occurred within 1 year of ideation onset (4), and approximately one-third of those who contemplate suicide will eventually also make a suicide attempt (5). Although SI does not appear to be more prevalent among veterans than among the general population (6, 7), when contemplations of suicide are translated into suicide attempts, these are more likely to result in death among veterans than among non-veterans (8). Investigating the underlying mechanisms of veteran SI is then imperative.

One major factor that puts veterans at risk for suicide is the emotional distress that military experiences foster [e.g., Ref. (9)]. The burden of coping with the aftermath of war, and particularly the pivotal role that posttraumatic stress disorder (PTSD) symptomatology plays in such coping efforts, may be cardinal for understanding the process linking war experiences to subsequent SI [e.g., Ref. (10)]. Former prisoners of war (ex-POWs) may be of particular interest in this respect because of the unique features of their trauma, which may implicate SI.

### War Captivity, Its Aftermath, and Subsequent SI

War captivity is one of the most severe and malicious experiences known to man [e.g., Ref. (11)], and therefore it is considered to be one of the most traumatic (12). Captors often go to great lengths to break the captive’s spirit, utilizing multiple assaults on the individual’s physical and mental integrities, including torture, humiliation, deprivation, solitary confinement, and capricious cruelty. Studies consistently reveal that ex-POWs exhibit worse postraumatic stress symptoms (PTSS) and PTSD than non-captive combat veterans [e.g., Ref. (13)]. Moreover, compared with non-captive combat veterans, ex-POWs exhibit more chronic and protracted PTSS, greater levels of a late-onset of symptom manifestation, and fewer indicators of symptomatic resilience or recovery (14).

Among victims of war incarceration, SI may initially appear during captivity [e.g., Ref. (15)] and might persist or reappear following repatriation [e.g., Ref. (16, 17)]. Surprisingly, although one may expect the effects of trauma to subside over time, and thus also expect a decline in SI, research indicates that ex-POWs evince an *increase* rather than a decrease in SI as time progresses, and this increase is steeper than among non-captive veterans (18). Notwithstanding, the investigation of long-term SI among this population is scarce, and the underlying mechanisms of this phenomenon remain largely uninvestigated. In this study, we worked toward filling this gap.

### Loneliness, SI, and PTSD

At the center of this study is the interplay between PTSD, on the one hand, and loneliness, which is typically defined as a cognitive discrepancy between the quantity or quality of relationships one perceives as having and those that are desired [e.g., Ref. (19, 20)], on the other hand. Both factors have been theoretically (21) and empirically associated with SI. The relation between PTSD and subsequent veteran SI has been demonstrated in several studies [e.g., Ref. (10, 22–24)], as was the relation between veterans’ SI with increased combat exposure, stigma, barriers to care, and decreased perceptions of social support (25).

The link between loneliness and SI is demonstrated in several contemporary models of suicide [e.g., Ref. (26–28)] and has been the subject of numerous empirical studies [for reviews, see Ref. (28, 29)]. Of particular note is Joiner’s interpersonal–psychological theory of suicide (28). According to this theory, the two primary contributors to SI are the subjective perception that one is a burden on his or her surrounding, and a sense of thwarted belongingness, which is constituted by loneliness. The theory’s potential utility in understanding veteran suicide has been gaining prominence [e.g., Ref. (30, 31)]. For instance, in a recent study among soldiers who had attempted suicide, Bryan and Rudd (9) found that over 62% of the sample felt lonely, isolated or abandoned 24 h before their suicide attempt. In the current study, we drew on this growing body of knowledge as our point of departure. However, we also diverged from its theoretical presuppositions in one important aspect.

While Van Orden et al. (28) view loneliness as a constituent of thwarted belongingness, with an emphasis on the latter, we view thwarted belongingness as one of several potential constituents of loneliness. Placing the emphasis on loneliness rather than belongingness, we underscore the need to view the sense of isolation that may beget veterans in a broader sense. A broader scope may enable the inclusion of modes of isolation that go beyond the lack of belongingness when considering post-war SI. The necessity of expanding the scope of investigation from thwarted belongingness to loneliness becomes clear as the multifariousness and polymorphic nature of the loneliness construct and the unique features of veterans’ and ex-POWs’ loneliness experiences are addressed.

### The Multifariousness of Loneliness and Its Qualities among Veterans

Drawing on loneliness’ cognitive conceptualization presented above, the experience is typically considered as synonymous with *perceived social isolation*. It is under this conceptualization that loneliness’ detrimental ramifications and clinical significance have been established (32, 33), including its association with SI [e.g., Ref. (34, 35)]. Nevertheless, loneliness is multifarious and polymorphic in nature and may manifest itself in different forms [e.g., Ref. (36, 37)]. For instance, loneliness may imply a perceived absence of care, assistance, empathy, intimacy, or any other provision that a meaningful relationship may offer (38, 39). The different sources of loneliness may be implicated in distinct psychopathologies and suicide outcomes (40), and thus its specific form in any given context must be taken into consideration.

Studies suggest that military loneliness is different from civilian loneliness (41), and that veteran loneliness is different still (42, 43). Specifically, combat veterans may feel that they belong in the military, where their capabilities are valued, but at the same time feel alienated and estranged from civilian society [e.g., Ref. (44–46)]. Furthermore, veterans may feel lonely in the sense that no one back home shares their experience or can understand what they have been through—neither family and friends, nor society at large (42, 43). This phenomenon has been conceptualized as “experiential loneliness,” connoting the sensation of being undesirably alone with one’s experiences. In a similar vein, research has shown that often traumatized veterans’ most pressing reintegration challenges include interpersonal difficulties, particularly in overcoming the challenge of confiding or sharing personal thoughts and feelings with others, keeping up non-military friendships, and belonging in “civilian” society (47). Nevertheless, a lack of belongingness is but one of several facets of veterans’ stratified experience of loneliness (42, 48).

Captivity may result in even greater degrees of loneliness than combat. First, isolation is often an inherent part of captivity. POWs spend protracted periods of time in solitary confinement, awaiting the unknown while anticipating unavoidable torture (49). Furthermore, a large portion of the traumatic experience of captivity occurs in the interpersonal domain. For instance, Laub and Auerhahn (50) underscore the experience of *failed empathy*—a person’s capacity to ignore the suffering of another and deliberately inflict it—as a central element of torture that impedes interpersonal connections thereafter. According to betrayal trauma theory (51, 52), interpersonal betrayals of basic human conduct exacerbate the effect of traumatic experiences and further hinder survivors’ capacity to trust others. Thus, not only is the POWs’ trauma typically considered to be more severe than that of non-captive combat veterans but due to the interpersonal aspects of captivity ex-POWs are also susceptible to additional interpersonal impediments, particularly insecure attachment orientations and loneliness (53–55). Similarly, recent research among incarcerated populations has indicated that past traumas may be implicated in more loneliness and less perceived social support (56).

This may be particularly the case with PTSD, the symptoms of which may include “feelings of detachment or estrangement from others” [(57), p. 272]. Indeed, Veterans’ PTSD has been found to hinder intimate relations [e.g., Ref. (58)] and potentially impede interactions among family [e.g., Ref. (59, 60)] and friends (61). Among ex-POWs, impediments to marital satisfaction were significantly associated with PTSD after repatriation, and this association was mediated by the loneliness related to their PTSS (62). Nevertheless, to the best of our knowledge, the mutual effects of PTSD and loneliness in relation to SI have not been investigated in this population.

This dearth is especially pertinent due to the findings suggesting that the association between loneliness and PTSS may be reciprocal and bidirectional (63, 64). Particularly, this reciprocal relation may be rooted in the consistent finding that the lack of social support after a traumatic episode is among the strongest risk factors for the development of PTSD (65, 66). According to Rook (67), social support and loneliness are two sides of the same coin and must be studied together. Conversely, a study among combat veterans suggests that social support may mitigate PTSD only if it also counters the veterans’ loneliness (68). This suggests that loneliness may underlie PTSS as well as contribute to its maintenance (64). Investigating the interplay between captivity trauma, loneliness, and PTSS may therefore be pertinent when trying to understand the underpinnings of ex-POWs’ SI. Altogether, it would seem that a more severe man-made traumatic experience (e.g., captivity vs. war) may be implicated in more severe PTSS or PTSD, more loneliness, and more SI. Therefore, in this study, we set out to examine the relations between these three factors.

### The Current Study

Drawing on our former investigations with this cohort (16, 62), we hypothesized that ex-POWs would evince higher PTSS, more loneliness, and more SI than non-captive veterans (H1). Furthermore, we hypothesized a sequential mediation model (H2), wherein the type of trauma (i.e., captivity vs. combat) was expected to contribute to the prediction of PTSS (H2a), which was expected to contribute to the prediction of the severity of loneliness (H2b), which was expected to contribute to the prediction of subsequent PTSS (H2c) and SI (H2d).

## Materials and Methods

### Participants and Procedure

This study is part of a longitudinal study among Israeli ex-POWs and comparable control veterans from the 1973 Yom Kippur War [for full details, see Ref. (14)], with assessments in 1991 (T1), 2003 (T2), and 2008 (T3). The ex-POWs fell captive during combat and were either held captive in Egypt for 6 weeks or in Syria for 8 months, approximately. The control group was matched for military assignment, unit and military duty as well as for scores on military performance prediction tests administered when first drafted. For this study, because loneliness was assessed only at T1, we used data only from that measurement and the following one (i.e., T1 and T2). At T1, assessment took place at a centrally located hospital, and at T2 questionnaires were administered at the participants’ homes or other locations of their choice. Participants’ informed consent was obtained. The ethics committees of the IDF and Tel Aviv University Institutional Review Board approved the study.

According to the Israeli Ministry of Defense, 240 soldiers in the IDF land forces were taken prisoner in the 1973 Yom Kippur War. Of the 240 ex-POWs, 164 ex-POWs participated at T1 (68.33% participation rate). At T2, 10 could not be located or refused to participate—4 were deceased and 6 could not participate due to mental deterioration—of the remaining 144, 103 agreed to participate at T2 (71.5% participation rate).

The control group consisted of 185 participants at T1 (out of 280 who were identified *via* the IDF computerized data banks). At T2, 41 of these could not be located, and 1 was deceased. Of the remaining 143 controls, 106 agreed to participate (74% participation rate).

### Handling Missing Data

To decide whether the data were missing at random (MAR), we conducted analyses of differences between these groups in all variables using Little’s Missing Completely at Random test (69). The analysis revealed that the data were not MAR, χ^2^ (45) = 76.076, *p* = 0.003, as the mechanism was shown to be related to the observed data (MAR). Under the erroneous assumption of MAR, missing data are proven to be better handled with maximum likelihood (ML), this method was therefore utilized in this study. Compared with conventional methods such as arithmetic mean, listwise, or pairwise deletion, and given the longitudinal design of this study, ML method is recommended as an optimal method for computing missing data to avoid bias [e.g., Ref. (70)]. This method uses all the available relevant data for each participant as well as over time because missing information can then be recovered from earlier or later waves. We, therefore, used the data of those who participated in the first wave as an anchor for data completion. The final sample after data completion included 163 ex-POWs and 185 controls.

### Measures

#### Suicidal Ideation

Suicidal ideation was assessed using two items from the Symptom Checklist-90 (71). The two items were as follows: (a) “*thoughts about ending your life*” and (b) “*thoughts about death*.” Participants were asked to indicate how frequently they experienced each symptom during the last 2 weeks on a 5-point scale (0 = not at all and 5 = very much). Due to the strong correlations between the two items (*r* = 0.56), we calculated the mean score of the two items as an SI index. The use of two items as indicators of SI is commonly utilized in the literature [e.g., Ref. (18, 72–74)]. Moreover, the use of a single item as well as two items for assessing SI has been compared with the utility of the gold standard for measuring SI [i.e., Scale for Suicide Ideation (75)] and was empirically established as a valid method of SI assessment (76).

#### PTSD and PTSS

Posttraumatic stress disorder inventory (PTSD-I; 77, 78) was used to measure PTSS. This instrument is based on the PTSD criteria in the DSM-IV (79), which was the standard at the time the research commenced. The questionnaire consists of 17 statements describing different expressions of PTSD following war. Items comprised of following three subscales: intrusion, avoidance, and arousal. The scale was found to have high convergent validity when compared with diagnoses based on structured clinical interviews, reaching 85% agreement (77, 78). Respondents were required to rate each statement according to the frequency they experienced it during the last month. Ratings appear on a 4-point scale ranging from “never” to “very often.” The total score for the scale was computed based on the total number of symptoms endorsed. In this study, the PTSD-I was found to have high internal consistency [α(T1) = 0.90, α(T2) = 0.95].

#### Loneliness

UCLA Revised Loneliness Scale (80) was used to measure loneliness. The scale consists of 20 items—10 reflecting satisfaction with social relationships and 10 reflecting dissatisfaction. The questionnaire targets perceived social isolation and connectedness in three main domains: general or intimate isolation (e.g., “I feel isolated from others”), relational disconnectedness (e.g., “There are people who really understand me”), and collective disconnectedness [e.g., “I have a lot in common with the people around me” (36)]. Participants were asked to indicate how often they experienced these feelings on a 4-point Likert scale (0 = *not at all*, 3 = *very often*). The scale has high internal reliability (α = 0.94) and concurrent and convergent validity (80). The total score is the sum of all items after reversing the positively worded items (maximum score is 60). High scores reflect more feelings of loneliness. The scale possesses good psychometric properties, in both its English (80) and Hebrew [e.g., Ref. (64)] versions. In this study, high internal consistency was found (α = 0.88).

## Results

### Demographics and PTSD

Former prisoners of war and controls did not differ in age, education, or income. At T2, the mean age of the participants was 53.4 (SD = 4.4), and mean years of schooling was 13.94 (SD = 3.45). Regarding income, 17% reported their income as lower than the average, 24% as average, 38% as slightly higher than average, and 21% as much higher than average. Significantly more ex-POWs (*N* = 23 of 99, 23.2%) met the DSM-IV symptom criteria for PTSD 30 years after the Yom Kippur War than non-POW controls (*N* = 4, 3.8%) (*N* = 205, χ^2^ = 16.70, df = 1, *p* < 0.001).

To assess the differences between ex-POWs and controls, we conducted *t*-test analyses for independent groups. As hypothesized (H1), results indicated that the ex-POW group scored significantly higher than controls on SI at T2 (M = 0.75, SD = 0.84, and M = 0.30, SD = 0.44, respectively; *t*(237) = 6.15, *p* < 0.001), loneliness at T1 (M = 18.79, SD = 10.43, and M = 15.80, SD = 9.59, respectively; *t*(346) = 13.27, *p* < 0.01) as well as PTSS at T1 (M = 3.48, SD = 4.50, and M = 1.85, SD = 2.90, respectively; *t*(269) = 3.98, *p* < 0.001) and T2 (M = 9.01, SD = 5.01, and M = 3.80, SD = 3.71, respectively; *t*(295) = 10.90, *p* < 0.001).

### Sequential Mediational Model

To test our hypothesized mediation model, we conducted a sequential mediational model assessment using SPSS macro PROCESS [Model 6 (81)] with 95% bias corrected confidence interval based on 5,000 bootstrap samples. Study group (ex-POWs = 1, controls = 0) served as the independent variable. PTSS at T1, loneliness at T1, and PTSS at T2 served as the mediators; SI at T2 was the outcome variable. Throughout the analysis, we controlled for the effect of SI at T1 on SI at T2.

**Figure 1:**
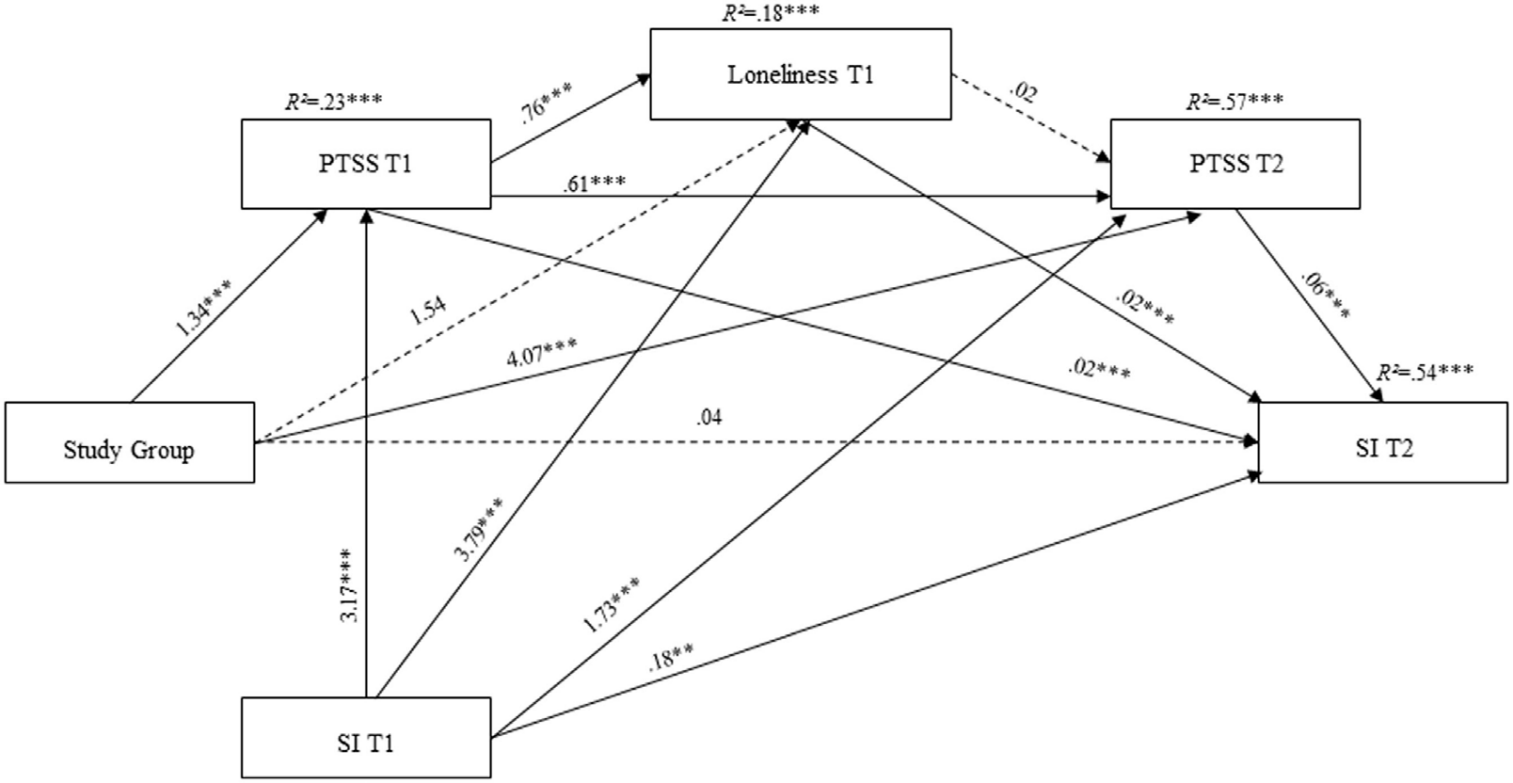
Statistical model of the sequential meditational model for the prediction of SI. Note: Study group is a dummy-coded variable (0 = controls veterans; 1 = ex-POWs); PTSS, posttraumatic stress symptoms; SI, suicide ideation; ex-POWs, former prisoners of war; SI at T1 is a covariate; coefficients are unstandardized; dashed lines represent non-significant paths; *p < 0.05, **p < 0.01, and ***p < 0.001.

As hypothesized (H2), the analysis yielded a significant model, *F*(5, 343) = 115.53, *p* < 0.001, *R*^2^ = 0.57 (see Figure 1), wherein the direct effect of captivity on SI at T2 was not significant when all of the mediators and hypothesized indirect effects were entered into the model. Notably, three indirect effects were found to be significant (see Table 1). First, we found a single 1-step mediation, wherein captivity indirectly affected PTSS at T2, which was then related to higher SI at T2. In other words, ex-POWs’ higher levels of PTSS, compared with controls, were related to increments in SI over time. In addition, we found two 2-step mediations. Captivity indirectly affected SI at T2 *via* PTSS and loneliness at T1. In other words, among ex-POWs, higher PTSS at T1 predicted higher loneliness cross-sectionally; loneliness predicted an increase in SI at T2. In the second 2-step mediation, captivity indirectly affected SI at T2 *via* PTSS at T1 and T2. Meaning that among ex-POWs, there were higher levels of PTSS at T1 that resulted in higher PTSS at T2; this was related to higher SI at T2. The remaining indirect effects were non-significant (Table 1). The ratio of indirect effects to total effects supported the results, thus indicating that the indirect effects identified in the analysis contributed significantly to the entire total effect.

**Table 1:** Bootstrapped point estimates for direct and indirect effects for predicting suicidal ideation.

		*b* (se)	95% Cl	Ratio of indirect effect to total	
	Direct effect	0.04 (0.06)	−0.0772, 0.1644		
Step 1	Indirect effects *via*				
	PTSS T1	0.03 (0.02)	−0.0075, 0.0839	0.07 (0.06)	−0.0181, 0.2042
	Loneliness T1	0.03 (0.02)	−0.0042, 0.0713	0.06 (0.04)	−0.0107, 0.1629
	**PTSS T1**	0.25 (0.06)	0.1512, 0.3675	0.58 (0.11)	0.3770, 0.8157
Step 2	**PTSS T1 and loneliness T1**	0.01 (0.00)	0.0080, 0.0401	0.05 (0.02)	0.0207, 0.0911
	**PTSS T1 and PTSS T2**	0.05 (0.02)	0.0278, 0.0948	0.13 (0.03)	0.0738, 0.2054
	Loneliness T1 and PTSS T2	0.00 (0.00)	−0.0011, 0.0096	0.00 (0.00)	−0.0026, 0.0215
	PTSS T1, loneliness T1, and PTSS T2	0.00 (0.00)	−0.0011, 0.0048	0.00 (0.00)	−0.0026, 0.0101

*PTSS, posttraumatic stress symptoms; T1, 1993, 18 years after the war; T2, 2003, 30 years after the war*.

*Boldface indicates significant pathways*.

## Discussion

In a previous study, we found that captivity was linked to SI *via* PTSS (18). Building on this, we set out to investigate whether this effect may be better understood when the interplay between ex-POWs’ PTSS and their post-repatriation loneliness are considered (H2). Results mostly confirmed our hypotheses, indicating that not only was captivity implicated in higher rates of SI, compared with war participation, but also the direct relation between captivity and SI at T2 was ostensibly nullified (i.e., non-significant) once PTSS and loneliness were taken into account. Specifically, we found indirect paths between captivity and SI through PTSS at T1 (18 years after the war), PTSS at T2 (30 years after the war), and the stability of PTSS across these two time points. We found an additional indirect path running through loneliness that was related to PTSS at T1. Nevertheless, our hypothesis that the link between captivity and subsequent SI would be explained by variations in loneliness, which are directly related to the type of trauma (i.e., captivity vs. combat), was not supported by the findings. In addition, our hypothesis that the link between captivity and subsequent SI would be explained by the link between loneliness and subsequent PTSS was not supported by the findings.

The finding that PTSS play a pivotal role in veterans’ posttraumatic SI is consistent with previous studies [e.g., Ref. (10, 23)]. Furthermore, the finding that ex-POWs exhibited more SI than non-captive veterans as a result of their posttraumatic reactions, supports the argument that different traumatic experiences give rise to different posttraumatic reactions (82). Extreme man-made traumas, such as captivity and torture, may be implicated in worse suicidal behaviors and ideations due to the nature of the precipitating event and its implications both for symptom manifestation and social connectedness. This finding is also consistent with studies indicating that interpersonal traumas are implicated in worse psychological consequences [e.g., Ref. (83)], less favorable interpersonal bonds [e.g., Ref. (84)], more suicide attempts [e.g., Ref. (85)], and higher degrees of SI [e.g., Ref. (86)] than non-man-made traumas (e.g., natural disasters).

The main contribution of this study, however, is that loneliness may play an important role in ex-POWs’ SI. This finding adds further support for one of the primary tenets of the interpersonal–psychological theory of suicide (27, 28) and provides further evidence for its application to veteran populations (31). That is, these findings imply that in order to understand post-captivity SI it may be paramount to consider the impediments to interpersonal connectedness, to which both captivity and subsequent PTSS may give rise.

Notably, the analysis revealed that SI at T2 was not explained by variances in loneliness that relate to variances in trauma type (i.e., captivity and non-captivity). Rather, the impact of trauma type on SI was explained only *via* its implication in PTSS, and the effect that PTSS had on loneliness. To understand this sequential effect, it is important to realize that loneliness is not merely another burdening experience but may also inhibit adaptive coping. This is because loneliness may entail maladaptive cognitions concerning other people and their intentions. Studies across phylogeny, underscore that loneliness is associated with increased awareness of social cues of potential rejection (32, 87, 88) and may be associated in reduced perceptions of social support after trauma (56). Applied to this study, it may be suggested that ex-POWs suffer from more PTSS than non-combatants, which is a burden by its own merit. Yet, due to the interpersonal nature of captivity trauma, ex-POWs often endorse maladaptive approaches toward interpersonal relationships (53, 54); and thus may be less likely to perceive others as potential support resources. The isolation embedded in the conviction that they must cope alone may give rise to hopelessness, which itself is a strong predictor of SI [e.g., Ref. (89, 90)] as well as suicide (91).

Arguably, veterans are lonely first and foremost in the sense that their experiences are theirs alone and cannot be adequately articulated and shared with others (48). Conversely, veterans may perceive themselves as being inherently different than civilians on the basis of their experiences in the war (45, 92, 93), thus thwarting belongingness and fostering alienation upon homecoming (44). This may be the case for non-captive veterans and ex-POWs alike (43). It may also explain why the difference in loneliness rates between the two study groups, while evident, was not explained by the traumatic experience alone, but rather by the PTSS it fostered. Indeed, veterans may also be lonely in the sense that they are convinced that they must cope alone with their traumatic past and posttraumatic present (48). Hence, as the psychological burden accumulates with the manifestation of PTSS, they may become lonelier, as the current findings suggest. Ultimately, when traumatic and posttraumatic burdens must be carried alone, one may wish to escape, and death by suicide may seem like an appealing way out.

As a whole, the co-manifestation and interaction of PTSS, loneliness, and SI may be related to a phenomenon that runs like a thread through these three elements, namely, mental pain. PTSS are diagnostically acknowledged as being tormenting and debilitating (57). Loneliness is depicted as an unbearably painful experience and has recently been found to share the same neural infrastructure as physical pain (94). Finally, mental pain and the desire to avoid it are prominent factors in all comprehensive theories of suicide (95) and their role in SI initiation is all but a consensus. Put otherwise, the nucleus of ex-POWs’ current torment and pain is constantly traced back to the trauma and its aftermath: the nightmares, the intrusive thoughts, the hypervigilance, the incapacity to reintegrate into civilian society and the sense of alienation this fosters, the conviction that one must cope alone, and the desire to put an end to all of this *via* suicide, are all attributed to the same painful origin—the time in incarceration and the torture it entailed, which have ultimately hindered future perceptions of interpersonal relationships. Hence they all come together to explain ex-POWs’ posttraumatic SI.

### Clinical Implications

This bears several important clinical implications. First, the centrality of PTSS in posttraumatic SI suggests that ex-POWs who suffer from PTSS should be assessed for SI, and that preventing SI among this population may require addressing their PTSS in therapy, as other studies have previously suggested (10). Moreover, clinicians may do well to assess and treat interpersonal impediments among ex-POWs and strive to uncover whether these are associated with SI and suicide plans. It has been argued that preventing suicide involves an empathic understanding as to the circumstances that have brought the person to the edge, thus breaching the suicidal person’s loneliness (96). Since the detachment at hand may be first and foremost of an experiential nature (43), clinicians might wish to employ intersubjective approaches (97, 98), which are oriented toward apprehending the subjective experience of the trauma survivor as fully as possible; or interpersonal psychotherapy [e.g., Ref. (99)], which has been found to be efficacious for treating PTSD (100). These may increase ex-POWs’ capacities to draw on social resources as they cope with their posttraumatic aftermaths. Alternatively, it may be useful to initiate group-therapy interactions. Indeed, by connecting veterans with other veterans they come to learn that they are not alone and that there are others who are also dealing with the trials and tribulations germane to such traumas [e.g., Ref. (101)]. Moreover, the commonality found in groups (e.g., the mutual understanding of the traumatic and posttraumatic experiences) may somewhat alleviate veterans’ isolation as it relieves them of their burdening secrets (12). These interventions may ultimately reduce ex-POWs’ SI by addressing both the trauma and the loneliness at its infrastructure, and thus, hopefully reduce death by suicide among this population.

### Study Limitations and Future Directions

The findings in this study must be interpreted in the context of several limitations. First, the sole use of self-report measures, especially for the assessment of SI, may be subjected to reporting bias. Furthermore, the use of two items to assess SI, while common in the literature and deemed valid when compared with well-validated measures of SI (76), is nevertheless limited in its capacity to account for the richness and variability of the phenomenon. Future studies may do well to use better, and if possible objective, assessment tools.

Second, the long periods between measurements and between the first point of measurement and the end of the war, as well as the measurement of loneliness only in the first wave, may have impeded our ability to draw definitive conclusions concerning the causal and temporal relation between the study variables. Future longitudinal efforts must then be undertaken to further substantiate the preliminary yet important findings of this study. Specifically, future studies should investigate the causal relations between PTSD and loneliness, and between both phenomena and subsequent SI. More generally, since there is a striking dearth of loneliness focused trauma research, the finding that loneliness may add to the explained variance in SI beyond that which is explained by either the trauma or its subsequent psychopathology, may set the stage for more comprehensive investigations of this deleterious phenomenon and its contextualized posttraumatic manifestations.

## Ethics Statement

The ethics committees of the IDF and Tel Aviv University Institutional Review Board approved the study. All participants gave written informed consent before participation in the study.

## Author Contributions

JS was the main author and has conceptualized the study. LI preformed the statistical analyses and wrote the draft for Sections “Materials and Methods” and “Results.” YL-B assisted in the conceptualization of the study, finalizing the manuscripts, and identifying relevant literature. ZS is the primary investigator, head of the research lab, and the initiator of data collection and research funding. She also supervised the entire study and the writing of the manuscript.

## Conflict of Interest Statement

The authors declare that the research was conducted in the absence of any commercial or financial relationships that could be construed as a potential conflict of interest.
